# Associations between exercise capacity, p16^INK4a^ expression and inflammation among adult survivors of childhood cancer

**DOI:** 10.3389/fonc.2022.1014661

**Published:** 2022-11-08

**Authors:** Chelsea G. Goodenough, Matthew D. Wogksch, Mondira Kundu, Matthew Lear, Paul G. Thomas, Deo Kumar Srivastava, Zhaoming Wang, Gregory T. Armstrong, Melissa M. Hudson, Leslie L. Robison, Kirsten K. Ness

**Affiliations:** ^1^ Department of Epidemiology and Cancer Control, St. Jude Children’s Research Hospital, Memphis, TN, United States; ^2^ Department of Cell and Molecular Biology, St. Jude Children’s Research Hospital, Memphis, TN, United States; ^3^ Department of Pathology, St. Jude Children’s Research Hospital, Memphis, TN, United States; ^4^ Department of Immunology, St. Jude Children’s Research Hospital, Memphis, TN, United States; ^5^ Department of Biostatistics, St. Jude Children’s Research Hospital, Memphis, TN, United States; ^6^ Department of Oncology, St. Jude Children’s Research Hospital, Memphis, TN, United States

**Keywords:** cellular senescence, p16, inflammation, childhood cancer surivor, exercise capacity

## Abstract

**Background:**

Over 50% of childhood cancer survivors are exercise intolerant, with maximal aerobic capacities comparable to individuals decades older, suggesting early physiologic ageing. In addition, 36% of survivors are obese. Optimal exercise capacity provides a foundation to support daily function and healthy body habitus and is associated with benefits to cognition, cardiovascular health, and longevity. Cellular senescence and inflammation are key mechanisms that drive age-related disease, quantifiable as biomarkers in peripheral blood.

**Aims:**

This study aimed to evaluate associations between p16^INKa^, a biomarker of cellular senescence, and inflammation and exercise capacity among adult survivors of childhood cancer.

**Materials and methods:**

Eligible survivors were recruited from the St. Jude Lifetime (SJLIFE) Cohort Study. Exercise capacity was assessed by maximal oxygen uptake (VO_2_, ml/kg/min) obtained *via* cardiopulmonary exercise testing using a modified Bruce protocol. Body fat (%) was determined from dual energy x-ray absorptiometry (DEXA). Peripheral blood samples were used to evaluate log_2_ p16^INK4a^ mRNA expression, a biomarker of cellular senescence, and inflammation with high sensitivity C-reactive protein (hs-CRP) levels. Multivariable regression evaluated associations between p16^INK4a^, hs-CRP, body fat, and exercise capacity.

**Results:**

Participants included 185 five-year childhood cancer survivors (mean age 36.6 [range 20.1 - 55.7] years, 44% male, 77% non-Hispanic white, 53% leukemia/lymphoma). Compared to males, females had lower peak VO_2_ (mean ± SD, 22.5 ± 8.2 vs. 28.8 ± 7.7 ml/kg/min, p<0.01), higher p16^INK4a^ expression (9.6 ± 1.2 vs. 9.2 ± 1.2 fold, p=0.02), and hs-CRP concentration (5.9 ± 8.4 vs. 3.3 ± 3.9 mg/L, p=0.01). Among females (n=103), hs-CRP concentration (β -0.2, 95% CI -0.34 to -0.05, p=0.01) and p16^INK4a^ expression (β-5.32, 95% CI 10.42 to -0.22, p=0.04) were inversely associated and statistically significant with peak exercise capacity, with a significant interaction between p16^INK4a^ expression and body fat (β 0.15, 95% CI 0.02 to 0.28, p=0.03). Among males (n=82), p16^INK4a^ expression (β -1.01, 95% CI -2.14 to 0.12, p=0.08), and body fat (β -0.54, 95% CI -0.70 to -0.38, p<0.01) were inversely associated with peak exercise capacity.

**Conclusion:**

Inflammation and p16^INK4a^ expression, a biomarker of cellular senescence, are associated with lower exercise capacity in childhood cancer survivors, suggesting potential targets or outcome measures for interventions designed to prevent or remediate accelerated physiologic ageing in this population.

## Introduction

Significant advances in treatment of childhood cancers have contributed to five-year survival exceeding 85% (1, 2). However, childhood cancer survivors are at risk for adverse health outcomes associated with cancer treatment, including exercise intolerance. Exercise intolerance is the result of impairment of or poor integration of cardiovascular, autonomic, pulmonary, muscular, and neurosensory system function. Over 56% of survivors are exercise intolerant (VO_2_ peak <85% predicted) (3), with exercise capacities comparable to individuals’ decades older (4). Young adult survivors of childhood cancer with exercise intolerance have a 3.9-fold increased risk of mortality (3). Within this population, risk for exercise intolerance is highest among those exposed to cardiotoxic therapy such as anthracyclines and chest radiation (3). However, survivors not exposed are also at risk, suggesting that either the disease process or other systemic alteration such as inflammation or cellular damage also contribute to decline in exercise capacity.

Cellular senescence is the functional consequence of serious DNA damage (5), resulting in accumulation of cells unresponsive to growth stimuli. Although these cells appear to remain in a stable state of proliferation arrest, they are not benign. Senescent cells accumulate with age (6–10), secrete high levels of inflammatory cytokines, immune modulators, growth factors, and proteases, and are associated with an increased prevalence of age-related health conditions, including both subclinical inflammation and high fat and/or low lean body mass (11). Although senescent cells are largely undetectable in younger populations, p16^INK4a^ expression (mRNA), a biomarker of biologic ageing and indicator of senescent cells in older adults, is elevated in skin biopsies of young survivors of childhood cancer exposed to radiation (12).

p16^INK4a^ is an important tumor suppressor gene that prevents cells with damaged DNA from growing and dividing too rapidly (13). When expressed, p16^INK4a^ binds to and inactivates cyclin-dependent kinases (CDK4, CDK6), preventing the phosphorylation of retinoblastoma protein (pRB), halting cell cycle progression and initiating cellular senescence (14–16). Expression of p16^INK4a^ occurs in response to stress, such as DNA damaging radiation and chemotherapy, and is highly expressed in senescent cells (15). Thus, it is an excellent biomarker for cellular senescence (17). Given that children with cancer are exposed to cancer therapies capable of inducing DNA damage, and that accelerated physiologic ageing is evident in this population (18–20), it is possible that senescent cells, with their secretory properties, may contribute to the pathobiology of exercise intolerance.

Further, cellular senescence is also associated with abnormal body composition; senescent cells accumulate in white adipose tissue (21), increasing the release and circulation of senescence-associated secretory phenotype (SASP) (10, 22, 23). Unfortunately, children with cancer experience significant changes in body composition during treatment (24–28), with increased risk for both obesity and underweight that can persist into survivorship (28–30). Childhood cancer survivors also have poor dietary habits (31, 32), which may further influence adipose tissue senescence (33). Early accumulation of adipose tissue may be a reservoir for senescent cells and a source of inflammation (34), underlining the pathobiology of early onset of age-related chronic conditions in this population. Recent evidence from murine and human studies suggest that interventions resulting in clearance of cells expressing the p16^INK4a^ gene are capable of delaying the onset of, and attenuate existing, metabolic abnormalities (33) and age-related conditions (11, 35), perhaps defining a potential targets for intervention among survivors.

In this study, we measured p16^INK4a^ expression, a biomarker of biologic age and cellular senescence, and high sensitivity C-reactive protein (hs-CRP), a biomarker of inflammation, in young adult survivors of childhood cancer and evaluated the cross-sectional associations with body fat percent and exercise capacity. We hypothesized that p16^INK4a^ expression and hs-CRP levels would be higher among survivors with high body fat and low exercise capacity.

## Materials and methods

### Study population

Participants for this study were St. Jude Lifetime Cohort (SJLIFE) members, a retrospective cohort with prospective follow-up designed to evaluate childhood cancer survivors as they age. The study design and characteristics of the study population have been previously described (36–38). Briefly, cohort members were diagnosed with childhood cancer between 1962 and 2012 and treated at St. Jude Children’s Research Hospital. For these analyses, participants were at least 18 years old, 10 years from their primary diagnosis, had no evidence of cancer recurrence, and had previous chemotherapy exposure. Potentially eligible participants returning for a second clinical visit to evaluate frail health (including measures of exercise intolerance and body composition) were randomly recruited to provide a blood sample until we reached a powered sample size of 196 participants. Pregnant women or those with a current cancer diagnosis were excluded. Medical records were abstracted by trained personnel to collect demographic information, including age at assessment, sex, height (m), and weight (kg).

### Biomarkers

Cellular Senescence Expression of p16^INK4a^ mRNA was determined from CD3 T-lymphocytes processed from peripheral blood samples. Cells were isolated and enriched to >90% purity using RosetteSep™ Human T Cell Enrichment Cocktail (STEMCELL Technologies, Cambridge, MA). Total RNA was isolated from T-lymphocytes (ZR-96 Quick-RNA kit, Zymo Research, Irvine, CA), and reverse transcribed into cDNA using ImProm-II reverse transcription system (Promega Corp., Madison, WI). cDNA was reversed transcribed using Taqman^®^ quantitative reverse-transcription PCR (ThermoFisher Scientific, Waltham, MA) to determine p16^INK4a^ mRNA expression levels. Expression of p16^INK4a^ mRNA transcript was normalized to 18s ribosomal RNA (HS03003631, Applied Biosystems, ThermoFisher Scientific - US, Waltham, MA) as previously described (39, 40). Data were log transformed for analysis (log_2_).

Inflammation High sensitivity C-reactive protein (hs-CRP) concentration (mg/L) was determined from serum samples isolated from peripheral blood. Blood samples of 2ml were collected in serum preparation tubes and allowed to clot completely at room temperature. Samples were centrifuged at 1,000-2,000 x g for 10 minutes in a refrigerated centrifuge. Serum supernatant was separated from samples in 1ml aliquots into ARUP standard Transport Tubes and refrigerated until processed on a Quantitative Immunoturbidimetry assay (reference value ≤3.0 mg/L) (41).

### Outcomes

#### Exercise capacity

Exercise capacity was determined *via* cardiopulmonary exercise testing (CPET) on a treadmill using a modified Bruce protocol (42). A leg (n=4) or arm (n=7) cycle ergometer was substituted using a ramp protocol if a participant was unable to walk on a treadmill (lower extremity paralysis, amputations without prostheses, or poor balance). Continuous breath by breath analysis, using a metabolic cart (Ultima CardioO2; MCG Diagnostics, St. Paul, MN), was used to estimate attainment of VO_2_ peak. Blood pressure was measured during each stage of the protocol, and a continuous 12-lead electrocardiogram (ECG) monitored cardiac symptoms (43). Cardiopulmonary exercise testing (CPET) was terminated for safety before maximal exertion for signs of ischemia (>2 mm ST depression), frequent arrhythmias (bigeminy and trigeminy), hypertensive blood pressure (BP) response (250/115 mm Hg), symptoms (e.g. angina, shortness of breath, wheezing), or failure of heart rate (HR) to increase with increased exercise intensity. Immediately at test termination, participants were asked for peak rating of perceived exertion (44).

#### Body fat

Body fat (percent [%]) was determined with dual x-ray absorptiometry (DEXA) using a total body scanning mode (QDR 4500, software version 13.3:3; Hologic, Bedford, MA) (45, 46).

### Covariates

#### Smoking history

Participants self-reported their smoking history, and were classified as current, former, or never smokers.

### Statistical analyses

Descriptive statistics characterized demographic and diagnosis related variables (**Table 1**). Given that exercise capacity and body composition are influenced by sex, we stratified analysis by sex. Comparisons between male and female participants were made using χ2 statistics or two sample t tests as appropriate. Associations between smoking history, hs-CRP concentration, and p16^INK4a^ expression were evaluated using linear regression models. Smoking history was not associated with either hs-CRP concentration or p16^INK4a^ expression, and was not retained in final multivariate models. Separate multivariable linear regression models were used to evaluate associations between p16^INK4a^ expression or hs-CRP concentration, and exercise capacity (ml/kg/min). Smoking status was evaluated as a potential covariate. Models were stratified by sex and adjusted for body fat % and age at assessment. Two-way interactions between either p16^INK4a^ expression or hs-CRP concentration and body fat % were evaluated in each model. All statistical analyses were performed using SAS 9.4 (SAS Institute, Inc., Cary, NC).

**Table 1:** Demographic and primary cancer characteristics of survivors of childhood cancer.

Characteristic	All Participants (n=185)	Males (n=82)	Females (n=103)
**Race/Ethnicity, N (%)**			
Black	39 (21.1)	13 (15.6)	26 (25.2)
Hispanic	3 (1.6)	2 (2.4)	1 (1.0)
White	143 (77.3)	67 (81.7)	76 (73.8)
**Mean Diagnosis Age, years (SD)**	8.0 (5.8)	7.5 (5.3)	8.4 (6.1)
**Mean Age at Evaluation, years (SD)**	36.9 (8.0)	34.9 (8.4)	37.9 (7.7)
**Mean Survival Time, years (SD)**	28.7 (9.0)	27.8 (8.6)	29.9 (9.2)
**Smoking Status, N (%)**			
Current	21 (11.3)	9 (11.0)	12 (11.7)
Former	25 (13.5)	12 (14.6)	13 (12.6)
Never	139 (75.1)	61 (74.4)	78 (75.7)
**Primary Cancer Diagnosis, N (%)**			
Leukemia	80 (43.2)	49 (49.0)	31 (37.8)
Lymphoma	34 (18.4)	17 (17.0)	17 (20.7)
Sarcoma	18 (9.7)	10 (10.0)	8 (9.8)
Neuroblastoma	15 (8.1)	10 (10.0)	5 (6.1)
Wilms Tumor	13 (7.0)	8 (8.0)	5 (6.1)
Central Nervous System	10 (5.4)	4 (4.0)	6 (7.3)
Retinoblastoma	5 (2.7)	1 (1.0)	4 (4.9)
Other	10 (5.4)	4 (4.0)	6 (7.3)
**Treatment Type, N (%)**			
Chemotherapy only	89 (48.1)	36 (43.9)	53 (51.5)
Chemotherapy + Radiation	96 (51.9)	46 (56.1)	50 (48.5)
**Treatment Duration, mean (SD)**			
Chemotherapy (years)	1.6 (1.3)	1.5 (1.1)	1.7 (1.4)
Radiation (days)	14.5 (18.1)	16.4 (19.1)	13.0 (17.1)
**Chemotherapy Agent, N (%)**			
Vinca Alkaloids	141 (76.2)	66 (80.5)	75 (72.8)
Anthracyclines	136 (73.5)	61 (73.4)	75 (72.8)
Alkylating Agents	131 (70.8)	62 (75.6)	69 (67.0)
Corticosteroids	101 (54.6)	44 (53.7)	57 (55.3)
Methotrexate	96 (51.9)	41 (50.0)	55 (53.4)
Epipodophyllotoxins	91 (49.2)	42 (51.2)	49 (47.6)
Platinum Agents	39 (21.1)	19 (23.2)	20 (19.4)

## Results

### Characteristics of study population

**Figure 1:**
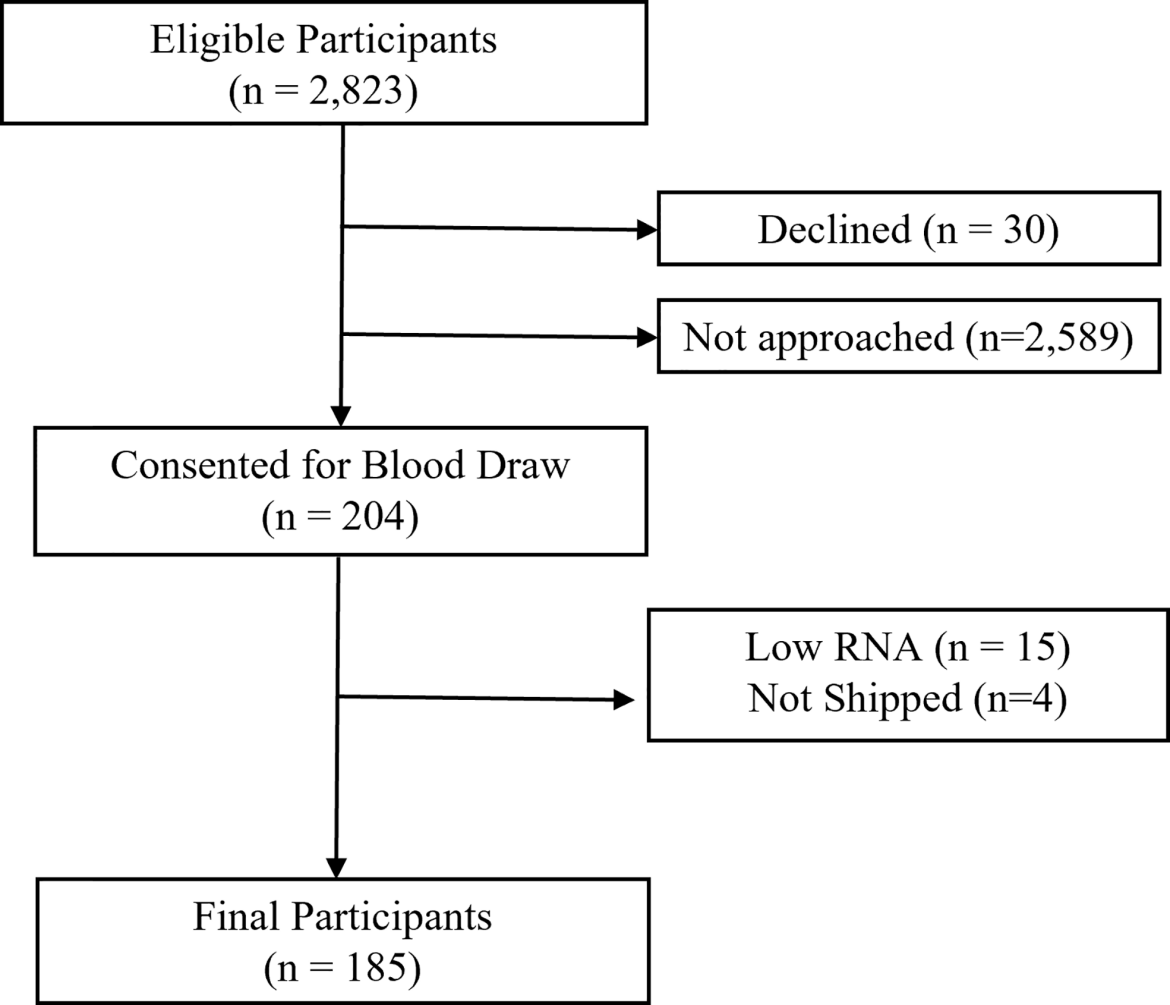
Participant flow.

Among 2,823 potentially eligible survivors, 234 were contacted to provide a blood sample. Among these, 30 (12.8%) declined participation. Of 204 samples collected, 15 (7.4%) did not pass quality control (low RNA yield), and 4 (2.0%) samples were not shipped, resulting in 185 participants with complete phenotype and biomarker data (**Figure 1**). Demographics of study participants are displayed in **Table 1**. On average, survivors were 36.6 years old (range 20.1 to 55.7 years) and 28.7 (± 9.01) years from diagnosis. Forty-four percent were male, 77% were non-Hispanic white, and 44% had a primary diagnosis of Acute Lymphoblastic or Myeloid Leukemia. Females were older than males at time of assessment (p<0.01).

### Exercise capacity and body fat

Female survivors had lower peak VO_2_ (mean (SD), 22.5 (8.2) vs. 28.8 (7.7) ml/kg/min, p<0.01) and higher body fat (39.2 (7.9) vs. 27.2 (8.2) %, p<0.01) compared to males (**Supplemental Figure 1**).

### Association between inflammation and exercise capacity

**Figure 2:**
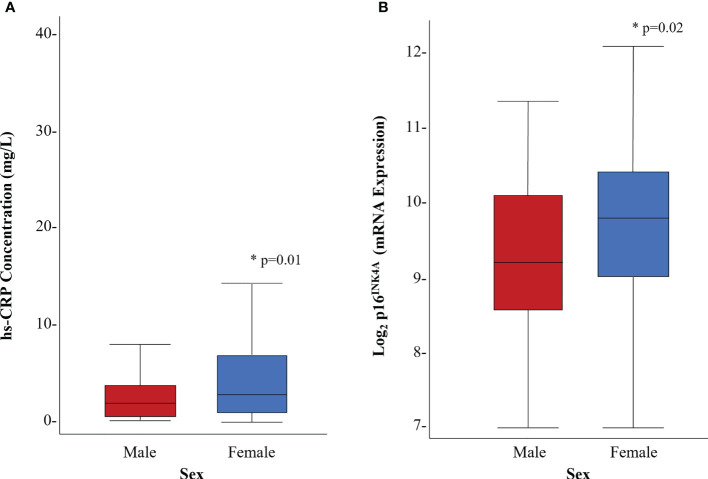
Distribution of biomarkers of inflammation and cellular senescence by sex. **(A)** hs-CRP concentration (mg/L) **(B)** p16 ^INK4A^ expression. * denotes statistical significance at p<0.05

**Figure 3:**
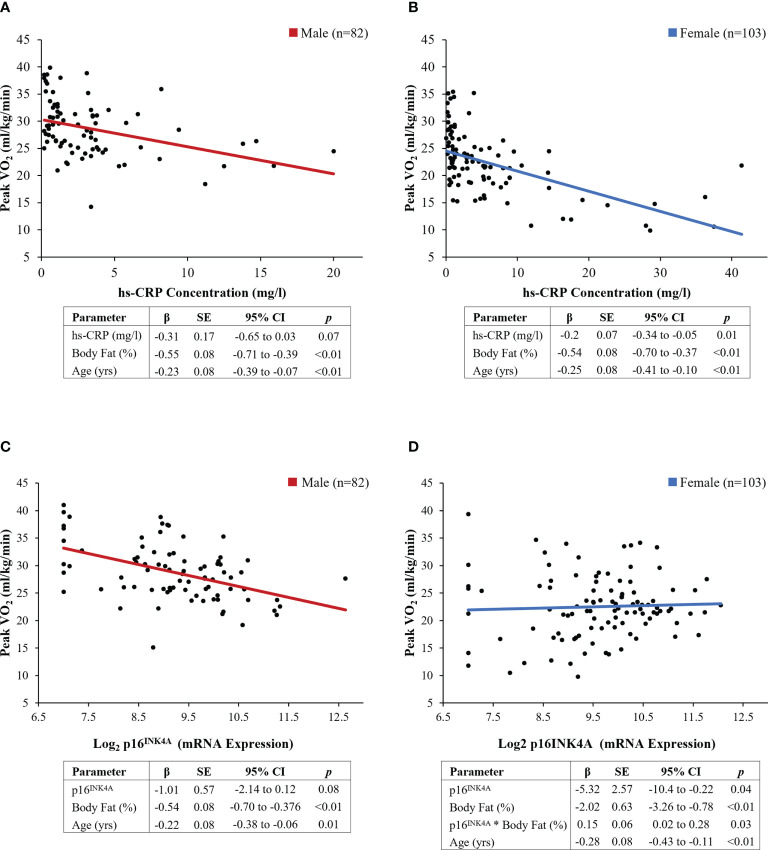
Results of sex-stratified multivariable models. **(A, B)** hs-CRP concentration (mg/L), **(C, D)** p16 ^INK4A^ expression. Regression lines represent the linear associations between individual biomarkers and exercise capacity, adjusted for covariates

Female survivors had higher hs-CRP concentrations (5.9 (8.4) vs. 3.3 (3.9) mg/L, p=0.01) than males (**Figure 2A**). The results of multivariable linear regression, stratified by sex, and adjusted for age (years), and body fat (%) are shown in **Figure 3**. Among the 103 female survivors, hs-CRP concentration (β -0.2, 95% CI -0.34 to -0.05, p=0.01), body fat (β -0.54, 95% CI -0.70 to -0.37, p<0.01), and age (β -0.25, 95% CI -0.41 to -0.10, p<0.01) were inversely associated with peak VO_2_ (ml/kg/min). Among the 82 male survivors, hs-CRP concentration (β -0.31, 95% CI -0.65 to 0.03, p=0.07), body fat (β -0.55, 95% CI -0.71 to -0.39, p<0.01), and age (β -0.32, 95% CI -0.39 to -0.07, p<0.01), were inversely associated with peak VO_2_ (ml/kg/min), though hs-CRP concentration did not achieve statistical significance.

### Association between p16^INK4a^ expression and exercise capacity

Female survivors had higher p16^INK4a^ expression (9.6 (1.2) vs. 9.2 (1.2) fold, p=0.02) compared to male survivors (**Figure 2B**). Multivariable linear regression models, stratified by sex, were used to evaluate the association between p16^INK4a^ expression and peak VO_2_ (ml/kg/min), adjusted for age (years) and body fat (%) (**Figure 3**). Among the 103 female survivors, p16^INK4a^ expression (β -5.32, 95% CI -10.42 to -0.22, p=0.04), body fat (β -2.02, 95% CI -3.26 to -0.78, p<0.01), age (β -0.28, 95% CI -0.43 to -0.11, p<0.01), and the interaction between p16^INK4a^ expression and body fat (β 0.15, 95% CI 0.02 to 0.28, p=0.03), were associated with peak VO_2_ (ml/kg/min). Among the 82 male survivors, p16^INK4a^ expression (β -1.01, 95% CI -2.14 to 0.12, p=0.08), body fat (β -0.54, 95% CI -0.70 to -0.38, p<0.01), and age (β -0.22, 95% CI -0.38 to -0.06, p=0.01) were inversely associated with peak VO_2_ (ml/kg/min), though p16^INK4a^ expression concentration did not achieve statistical significance.

**Figure 4:**
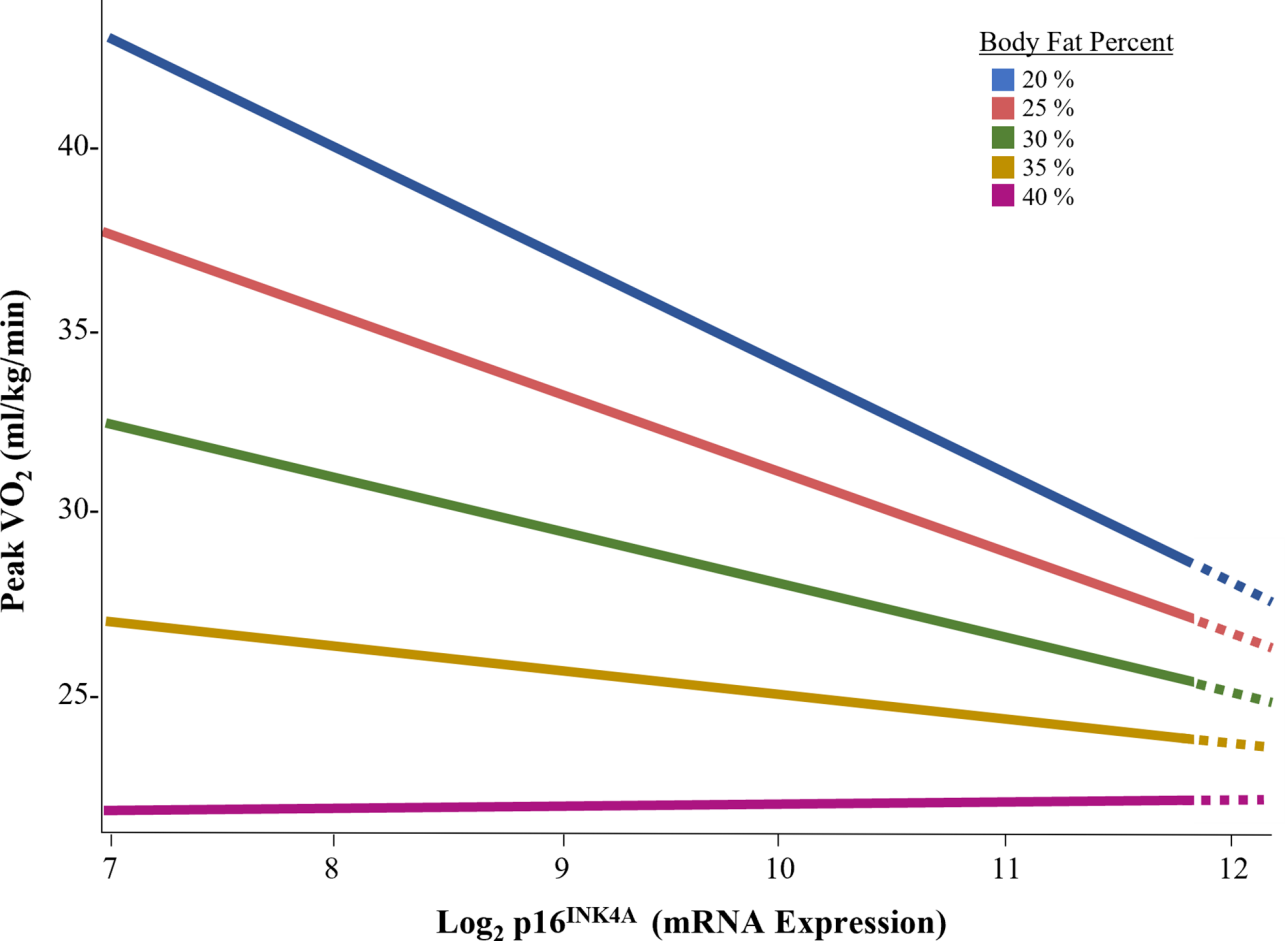
Association of p16^INK4A^ expression and exercise capacity by body fat percent in female survivors.

To explore the interaction effect of p16^INK4a^ expression and body fat on exercise capacity in females, data were sliced by progressing levels of body fat percent (**Figure 4**). Expression of p16^INK4a^ was inversely associated with exercise capacity at body fat percentages less than 35%.

## Discussion

Adult survivors of childhood cancer are at risk for exercise intolerance, a predictor of all-cause mortality (3). In this study, we found increased p16^INK4a^ and low-grade inflammation was associated with reduced exercise capacity. Among females, this association was only present among survivors with body fat percentages less than 35%. To our knowledge, this is the first study to demonstrate an association between a biomarker of cellular senescence, low-grade inflammation, and exercise capacity in childhood cancer survivors.

Expression of p16^INK4a^ is generally undetectable in children and younger adults (39). However, it is detectable in peripheral blood T-lymphocytes among older adults and among young survivors of childhood cancer exposed to radiation (12). More recently, Smitherman et al (47) showed evidence of p16^INK4a^ expression in young survivors of childhood, adolescent, and young adult cancers. They found elevated levels of p16^INK4a^ to be associated with frailty, an age-associated phenotype indicating reduced physiological reserve. These data and our findings support the hypothesis that p16^INK4a^ expression is present in other tissues (i.e. organs), and thus cellular senescence is potential mediator of physiologic deregulation in childhood cancer survivors.

Further, not only was elevated p16^INK4a^ expression and hs-CRP concentration associated with lower peak VO_2_ and exercise intolerance, but the mean VO_2_ peak among our survivors was similar to values in persons several decades their senior (4). This is concerning as poor exercise capacity is associated with early mortality (48, 49), future cardiovascular events (50), and reduced cognitive reserve (51). Early impairments in exercise capacity concomitant with a hallmarks of ageing suggest that VO_2_ may be a new biomarker capable of identifying survivors at greatest risk of early onset of chronic conditions and mortality.

The detection of these ageing biomarkers is not surprising given recent work that identified other hallmarks of ageing, including reduced physiologic reserve (19, 52), telomere attrition (53), altered DNA methylation patterns (54), and mitochondrial dysfunction (55). Cellular senescence is an important biological mechanism, and is a part of normal ageing. Inherently designed to guard against proliferation of damaged cells, senescent cells lose the capacity to replicate. As a result of cell cycle arrest, senescent cells secrete proteins, including growth factors and proteases that alter tissue structure and function, and cytokines and chemokines with pro-inflammatory properties. The SASP promote a state of subclinical inflammation, which results in tissue fibrosis and deterioration (56). A similar mechanism may be responsible for the early onset of reduced exercise capacity seen among young adult survivors of childhood cancer. Early exposure to DNA damaging agents may trigger early accumulation of senescent cells that is not completely reversible.

Senescent cells also accumulate dysfunctional mitochondria, capable of influencing SASP production (57). Our recent work found association between decreased mitochondrial copy number (mtDNAcn) and increased odds for sarcopenia (55). Impaired skeletal muscle oxidative phosphorylation is implicated in exercise intolerance among induvial with mitochondrial myopathies (58). It is possible that mitochondrial dysfunction is the pathobiological mediator between elevated p16^INK4a^ and hs-CRP levels in survivors with low exercise capacity.

Our data demonstrating elevated p16^INK4a^ expression and hs-CRP concentration in survivors with impaired exercise capacity indicates a potential intervention target, given evidence that p16^INK4a^ levels are modifiable, potentially with exercise (59, 60). In an animal model, Schafer et al (59) demonstrated an improvement in exercise capacity and a concomitant reduction in diet-induced p16^INK4a^ mRNA expression in rodents who exercised. Resistance training, although primarily associated with muscle mass and strength gains, also has the potential to clear accumulated senescent cells. Yang et al. noted significant gains in muscle mass and an associated rapid clearance of senescent cells from skeletal muscle tissue in young men following a bout of resistance training (60). Given that childhood cancer survivors respond to both aerobic and resistance training, with improved exercise tolerance (61–63) and strength and mass gains (64), following exercise, it is possible that either aerobic training and/or resistance training may contribute senescent cells clearance. Additional research to determine if exercise, including type, frequency, intensity, and duration of activity, can clear senescent cells and either prevent or delay the cellular ageing in survivors. Further, cellular senescence is targetable through nutraceuticals (65). Agents such as Dasatinib and flavonoids (Quercetin; Fisetin, available as nutritional supplements) interfere with the senescent pathway, with evidence of safety, tolerability, and ability to alleviate physical dysfunction in adults with chronic disease (66). Currently, we have an open-label intervention trial (NCT04733534) which aims to establish preliminary evidence of efficacy, safety, and tolerability of two senolytic regimens to reduce markers of cellular senescence and improve frailty in adult survivors of childhood cancer. Survivors with reduced exercise capacity may also benefit from senolytic agents either alone or in combination with lifestyle modifications.

In general population, overweight and obese individual have higher proinflammatory plasma profiles, specifically higher hs-CRP, than non-overweight or obese individuals (67). In our study, female survivors had significantly higher levels of body fat, concomitant with higher p16^INK4A^ expression, hs-CRP concentration, and lower peak VO_2_ (ml/kg/min) compared to males. Further, we noted a significant interaction between p16^INK4a^ expression and body fat among female survivors, suggesting that the effect of cellular senescence on exercise capacity may be masked in females who have excess body fat. In our study, at body fat % values less than 35%, p16^INK4a^ expression had a strong inverse association with exercise capacity. This association was not seen in females who had body fat % greater than 35% (68–70). Because adipose tissue is a harbor for senescent cells, is associated with reduced physical function, and is redistributed with ageing (71–73), it is possible that an evaluation of senescent cell expression in adipose tissue may have yielded different results. It is also possible that the burden of excess body fat is the primary driver of exercise capacity in females who are overweight or obese. Regardless, the influence of an interaction between body fat and biomarkers of ageing on exercise capacity are compelling and deserve further investigation.

The results of this analysis should be interpreted in the context of study limitations. Our population was small, and childhood cancer diagnoses were not evenly represented in the sample; almost half of the survivors had a history of childhood leukemia, followed by less than 20% with a history of lymphoma. While our study is limited by the use of a single biomarker of cellular senescence, our findings of detectable p16^INK4a^ mRNA expression levels higher in young adult childhood cancer survivors than in persons of similar age in the general population (39), combined with the presence of low grade inflammation in our population, signifies that cellular senescence is a potential pathobiological mechanism for premature physiologic ageing in survivors of childhood cancer, contributing to exercise capacities comparable to adults decades older. Further, we did not compare p16^INK4a^ and hs-CRP levels to individuals without a history of cancer. However, our mean hs-CRP value was higher than seen in a general population (41), suggesting a potential ongoing inflammatory milieu in this population. Liu et al (39) evaluated p16^INK4a^ expression in peripheral blood T-cells in healthy adults, much older than our participants (age [years], median [range], 47 [18-76] vs. 36.3 [20.1-55.7]), who were also overweight (body mass index (BMI) [kg/m^2^], median [range], 26.5 [9.4-55.1] vs. 29.6 [16.5-57.4] kg/m^2^). However, our participants had higher expression of p16^INK4a^ than healthy persons in the paper by Liu et al (39) (**Figure 2B**) (mean log_2_ p16^INK4a^ mRNA expression 9.4 vs. 5.5). We suscept that individuals without a history of cancer would have lower levels of both biomarkers given their lack exposure to radiation and chemotherapeutic agents capable of inducing DNA damage. Additionally, our analysis was cross-sectional, and thus we are limited in the ability to determine the direction of causality between p16^INK4a^ and exercise intolerance, and other participant characteristics, such as body composition. However, expression of p16^INK4a^ was not independently associated with BMI (p=0.08) or body fat (p=0.07), which is consistent with findings by Liu et al. (39) Further, we previously showed that over 50% of childhood cancer survivors are exercise intolerant, at BMIs comparable to healthy community controls (3), thus challenging that high adiposity is the true etiology of senescence in our survivors. More likely, adiposity and the accumulation of senescent cells in adipose tissue is additive to the pathobiology of exercise intolerance in this population. However, adiposity is a potential source of senescence etiology and further investigations into its interplay with biologic ageing, cellular senescence, and inflammation is warranted in the survivor population.

## Impact statement

Cellular senescence is implicated with advancing age and the onset of chronic condition and disease. Over 50% of young childhood cancer survivors are exercise intolerant, with maximal aerobic capacities comparable to individuals decades older, suggesting early physiologic ageing. In our study, biomarkers of cellular senescence and inflammation were associated with lower exercise capacity, which was further mediated by body fat in female survivors. To our knowledge, this is the first study to demonstrate an association between p16^INK4a^ expression, low-grade inflammation, and exercise capacity in childhood cancer survivors. Our study contributes to growing body of evidence of accelerated ageing among childhood cancer survivors. Further, it highlights that interventions designed to improve exercise capacity and/or body composition have potential to remediate the accelerating ageing phenotype and early onset of chronic conditions seen among adult survivors of childhood cancer.

## Data availability statement

The datasets analyzed for this study can be found in the St. Jude Cloud (https://www.stjude.cloud) (74).

## Ethics statement

The studies involving human participants were reviewed and approved by St. Jude Children’s Research Hospital Institutional Review Board. The patients/participants provided their written informed consent to participate in this study.

## Author contributions

CGG, MK, DKS, and KKN contributed to the conception and design of the research, analyzed, and interpreted the data. CGG, MDW, ML, and ZW performed the data acquisition and processing. CGG drafted the manuscript. All authors contributed to the article and approved the submitted version.

## Funding

This work was supported by grants provided by the National Cancer Institute (CA195547, MMH and KKN; CA174851 KKN), the Cancer Center Support Grant (CA21765, CRR), and the American Lebanese Syrian Associated Charities (ALSAC).

## Conflict of interest

The authors declare that the research was conducted in the absence of any commercial or financial relationships that could be construed as a potential conflict of interest.

## Publisher’s note

All claims expressed in this article are solely those of the authors and do not necessarily represent those of their affiliated organizations, or those of the publisher, the editors and the reviewers. Any product that may be evaluated in this article, or claim that may be made by its manufacturer, is not guaranteed or endorsed by the publisher.
