# Supplementary material for: Associations between exercise capacity, p16INK4a expression and inflammation among adult survivors of childhood cancer
Source: Front Oncol. 2022 Nov 8;12:1014661. doi: 10.3389/fonc.2022.1014661 (PMC9679643; doi:10.3389/fonc.2022.1014661)

Table S1. Distribution of blood biomarkers, body fat and exercise capacity among participants

**Outcomes**	**ALL Participants**<br>Mean (SD)	**Males**<br>Mean (SD)	**Females**<br>Mean (SD)	**Male vs Female**<br>***P***
$p16^{INK4A}$ Expression	9.4 (1.2)	9.2 (1.2)	9.6 (1.2)	0.02
hs-CRP Concentration (mg/l)	4.8 (6.9)	3.3 (3.9)	5.9 (8.4)	0.01
Exercise Capacity (ml/kg/min)	25.3 (8.5)	28.8 (7.7)	22.5 (8.2)	<0.01
Body Fat (%)	33.9 (10.0)	27.2 (8.2)	39.2 (7.9)	<0.01

Supplement Figure 1. Distribution of peak exercise capacity and body fat by sex. A) peak exercise capacity (ml/kg/min), B) Body fat (%).

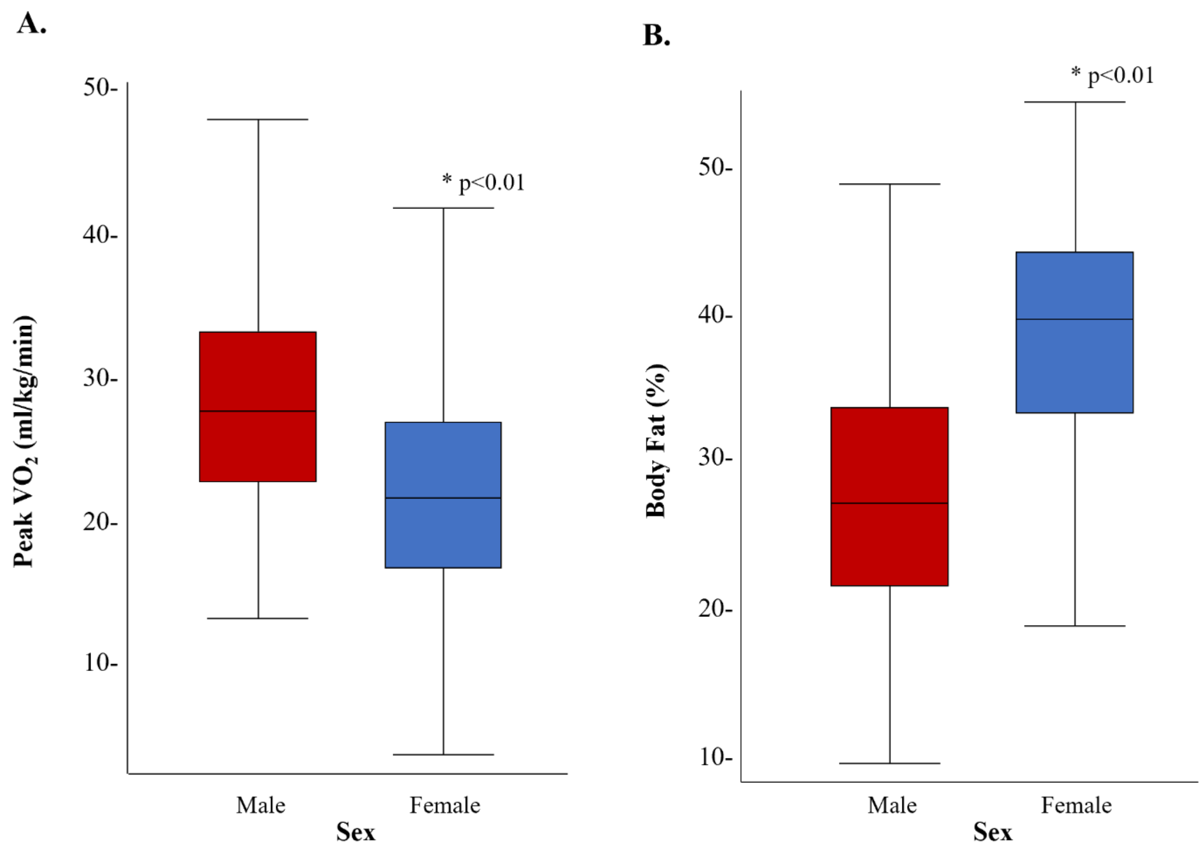

Supplement: Supplementary file 1 [file DataSheet_1.pdf]
